# 6-(4-Bromo­phen­yl)-2-eth­oxy-4-(2,4,6-trimethoxy­phen­yl)nicotinonitrile^1^

**DOI:** 10.1107/S1600536809043943

**Published:** 2009-10-31

**Authors:** Suchada Chantrapromma, Hoong-Kun Fun, Thitipone Suwunwong, Mahesh Padaki, Arun M. Isloor

**Affiliations:** a Crystal Materials Research Unit, Department of Chemistry, Faculty of Science, Prince of Songkla University, Hat-Yai, Songkhla 90112, Thailand; b X-ray Crystallography Unit, School of Physics, Universiti Sains Malaysia, 11800 USM, Penang, Malaysia; c Department of Chemistry, National Institute of Technology–Karnataka, Surathkal, Mangalore 575 025, India

## Abstract

In the asymmetric unit of the title nicotinonitrile derivative, C_23_H_21_BrN_2_O_4_, there are two non-planar independent mol­ecules. The central pyridine ring makes dihedral angles of 9.05 (7) and 77.06 (7)°, respectively, with the 4-bromo­phenyl and 2,4,6-trimethoxy­phenyl rings in one mol­ecule, whereas the corresponding values are 5.96 (7) and 82.37 (7)° in the other. All the three meth­oxy groups are essentially in the plane of the attached benzene ring [C—O—C—C angles = 2.99 (19), 4.8 (2) and −6.2 (2)° in one mol­ecule, and 2.69 (18), 176.73 (15) and 1.3 (2)° in the other]. The eth­oxy group is slightly twisted in one mol­ecule [C—C—O—C = 173.84 (12)°], whereas it is coplanar with the pyridine ring in the other [C—C—O—C = −177.23 (13)°]. Weak intra­molecular C—H⋯N inter­actions generate *S*(5) ring motifs. In the crystal structure, the mol­ecules are linked by weak inter­molecular C—H⋯N and C—H⋯O inter­actions into a supra­molecular three-dimensional network in such a way that the nicotinonitrile units of neighboring mol­ecules are stacked in an anti­parallel manner along the *c* axis. The crystal is further stabilized by C—H⋯π inter­actions.

## Related literature

For bond-length data, see: Allen *et al.* (1987 ▶). For hydrogen-bond motifs, see: Bernstein *et al.* (1995 ▶). For the synthesis and applications of nicotinonitrile derivatives, see: Abdel-Aziz (2007 ▶); Borgna *et al.* (1993 ▶); Chantrapromma *et al.* (2009 ▶); Goda *et al.* (2004 ▶); Raghukumar *et al.* (2003 ▶). For the stability of the temperature controller used in the data collection, see: Cosier & Glazer (1986 ▶).
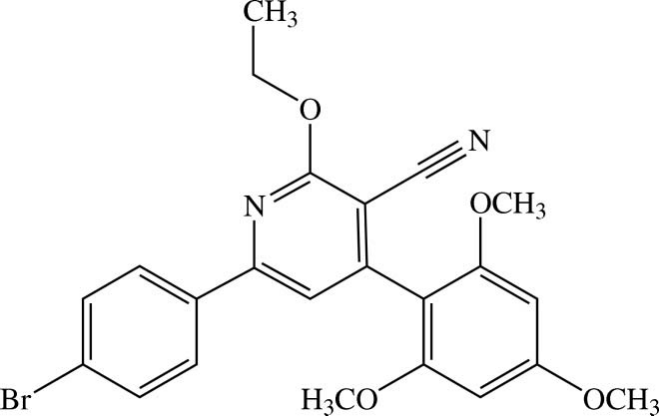


## Experimental

### 

#### Crystal data

C_23_H_21_BrN_2_O_4_
*M* *_r_* = 469.32
Monoclinic, 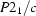

*a* = 14.1799 (2) Å
*b* = 18.0877 (3) Å
*c* = 16.6881 (2) Å
β = 95.081 (1)°
*V* = 4263.38 (11) Å^3^
*Z* = 8
Mo *K*α radiation
μ = 1.96 mm^−1^
*T* = 100 K
0.51 × 0.49 × 0.22 mm

#### Data collection

Bruker APEXII CCD area-detector diffractometer
Absorption correction: multi-scan (**SADABS**; Bruker, 2005 ▶) *T*_min_ = 0.437, *T*_max_ = 0.669
85168 measured reflections
18727 independent reflections
12072 reflections with *I* > 2σ(*I*)
*R*_int_ = 0.044

#### Refinement

*R*[*F* ^2^ > 2σ(*F* ^2^)] = 0.038
*wR*(*F* ^2^) = 0.095
*S* = 1.01
18727 reflections
549 parameters
H-atom parameters constrained
Δρ_max_ = 0.50 e Å^−3^
Δρ_min_ = −0.45 e Å^−3^

### 

Data collection: *APEX2* (Bruker, 2005 ▶); cell refinement: *SAINT* (Bruker, 2005 ▶); data reduction: *SAINT*; program(s) used to solve structure: *SHELXTL* (Sheldrick, 2008 ▶); program(s) used to refine structure: *SHELXTL*; molecular graphics: *SHELXTL*; software used to prepare material for publication: *SHELXTL* and *PLATON* (Spek, 2009 ▶).

## Supplementary Material

Crystal structure: contains datablocks global, I. DOI: 10.1107/S1600536809043943/is2473sup1.cif

Structure factors: contains datablocks I. DOI: 10.1107/S1600536809043943/is2473Isup2.hkl

Additional supplementary materials:  crystallographic information; 3D view; checkCIF report

## Figures and Tables

**Table 1:** Hydrogen-bond geometry (Å, °)

*D*—H⋯*A*	*D*—H	H⋯*A*	*D*⋯*A*	*D*—H⋯*A*
C1*A*—H1*AA*⋯N1*A*	0.93	2.45	2.7872 (19)	101
C1*A*—H1*AA*⋯O4*B*^i^	0.93	2.60	3.4813 (18)	159
C8*A*—H8*AA*⋯O2*B*	0.93	2.44	3.3391 (16)	162
C1*B*—H1*BA*⋯N1*B*	0.93	2.43	2.7751 (19)	102
C8*B*—H8*BA*⋯O2*A*	0.93	2.39	3.2889 (16)	162
C18*A*—H18*B*⋯O4*B*^i^	0.97	2.57	3.3360 (18)	136
C20*A*—H20*B*⋯O2*A*^ii^	0.96	2.58	3.2869 (17)	131
C20*B*—H20*E*⋯O2*B*^iii^	0.96	2.55	3.2169 (17)	126
C21*A*—H21*B*⋯O3*B*^iv^	0.96	2.57	3.154 (2)	119
C22*A*—H22*B*⋯N2*B*^iii^	0.96	2.58	3.479 (2)	155
C18*B*—H18*D*⋯*Cg*1^iv^	0.97	2.93	3.7798 (16)	147
C20*A*—H20*C*⋯*Cg*3	0.96	2.60	3.5075 (15)	157
C20*B*—H20*F*⋯*Cg*2	0.96	2.51	3.3845 (15)	152

Symmetry codes: (i) 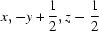; (ii) 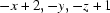; (iii) 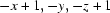; (iv) 
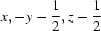
. *Cg*1, *Cg*2 and *Cg*3 are the centroids of the C7*A*–C11*A*/N1*A*, C12*A*–C17*A* and C12*B*–C17*B* rings, respectively.

**Fig. 1.**
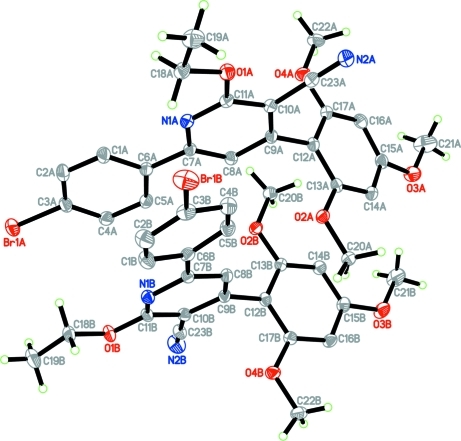
The molecular structure of the title compound, showing 50% probability displacement ellipsoids and the atom-numbering scheme. For clarity, aromatic H atoms are not shown.

**Fig. 2.**
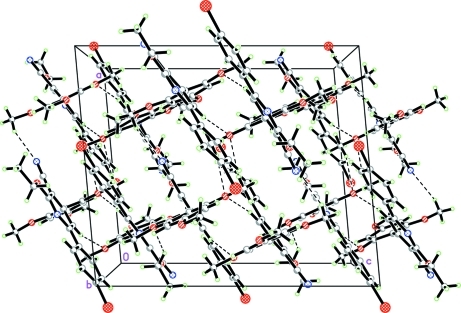
The crystal packing of the title compound viewed along the b axis, showing supramolecular three-dimensional network. Hydrogen bonds are shown as dashed lines.

## supplementary crystallographic information

### Comment

The pyridine ring is among the most common heterocyclic compound found in the naturally occurring heterocycles and in various therapeutic agents. The substituted pyridine derivatives have been claimed to have several biological activities (Borgna *et al.*, 1993; Goda *et al.*, 2004) and non-linear optical properties (Raghukumar *et al.*, 2003). The title nicotinonitrile derivative is a compound containing a pyridine ring which was synthesized by cyclization of chalcone derivative (Chantrapromma *et al.*, 2009) and malononitrile in order to be tested as antibacterial agents. It was tested against both Gram-positive bacteria *i.e. Staphyrococcus aureus*, *Bacillus subtilis*, *Enterococcus faecalis*, Methicillin-Resistant *Staphyrococcus aureus* and Vancomycin-Resistant *Enterococcus faecalis*, and Gram-negative bacteria *i.e. Pseudomonas aeruginosa*, *Salmonella typhi* and *Shigella sonnei*. Our results showed that the title compound has no antibacterial action against these pathogens, having the same results as its starting chalcone derivative (Chantrapromma *et al.*, 2009). Herein we report the crystal structure of the title compound (I).

There are two crystallographic independent molecules *A* and *B* in the asymmetric unit of (I) (Fig. 1) with slight differences in bond angles and in the conformation of the middle methoxy group in 2,4,6-trimethoxyphenyl unit between the two molecules. The molecular structure of (I), C_23_H_21_BrN_2_O_4_ is not planar. The central pyridine ring is nearly planar with the 4-bromophenyl ring with the dihedral angles of 9.05 (7)° [5.96 (7)° in molecule *B*] whereas is inclined to the 2,4,6-trimethoxyphenyl unit with the torsion angle of 77.06 (7)° [82.37 (7)° in molecule *B*] due to the steric effect between the methoxy and cyano groups. All the three methoxy groups are nearly co-planar to the attached benzene ring with the torsion angles C20–O2–C13–C14 = 2.99 (19)°, C21–O3–C15–C16 = 4.8 (2)° and C22–O4–C17–C16 = -6.2 (2)° in molecule *A* and the corresponding values are 2.69 (18), 176.73 (15) and 1.3 (2)° in molecule *B*. However these values show that the middle methoxy group is in different orientation in which it tilts to the methoxy group at C17 in molecule *A* but tilts to the methoxy group at C13 in molecule *B*. The ethoxy group in molecule *A* is slightly twisted with respect to the pyridine ring as indicated by the torsion angles C11–O1–C18–C19 of 173.84 (12)° and N1–C11–O1–C18 = 7.48 (19)° whereas it is co-planar in molecule *B* as shown by the corresponding values of -177.23 (13) and 0.12 (19)°. Intramolecular C1A—H1AA···N1A and C1B–H1BA–N1B interactions generate S(5) ring motifs (Bernstein *et al.*, 1995). The bond distances agree with the literature values (Allen *et al.*, 1987).

In the crystal structure (Fig. 2), the molecules are linked by intermolecular C—H···N and C—H···O weak interactions (Table 1) into a supramolecular three-dimensional network in such a way that the nicotinonitrile moiety of the neighbouring molecules are stacked in an antiparallel manner along the *c* axis. The crystal is further stabilized by C—H···π interactions (Table 1); *Cg*_1_, *Cg*_2_ and *Cg*_3_ are the centroids of C7A–C11A/N1A, C12A–C17A and C12B–C17B rings, respectively.

### Experimental

*E*-1-(4-Bromophenyl)-3-(2,4,6-trimethoxyphenyl)prop-2-en-1-one which was synthesized according to the previous procedure (Chantrapromma *et al.*, 2009) (0.57 g, 0.0015 mol) were added with continuous stirring to a freshly prepared sodium alkoxide (0.0014 mol of sodium in 100 ml of ethanol). Malononitrile (1.30 g, 0.02 mol) was then added with continuous stirring at room temperature until the precipitate was separated out. The resulting solid was filtered (yield 72%). Colorless block-shaped single crystals of the title compound suitable for *X*-ray structure determination were recrystalized from ethanol by the slow evaporation of the solvent at room temperature after several days (m.p. 423–424 K).

### Refinement

All H atoms were positioned geometrically and allowed to ride on their parent atoms, with d(C—H) = 0.93 Å for aromatic, 0.97 Å for CH_2_ and 0.96 Å for CH_3_ atoms. The *U*_iso_ values were constrained to be 1.5*U*_eq_ of the carrier atom for methyl H atoms and 1.2*U*_eq_ for the remaining H atoms. A rotating group model was used for the methyl groups. The highest residual electron density peak is located at 0.69 Å from C16B and the deepest hole is located at 0.41 Å from Br1B.

### Crystal data

**Table:**

C_23_H_21_BrN_2_O_4_	*F*(000) = 1920
*M**_r_* = 469.32	*D*_x_ = 1.462 Mg m^−^^3^
Monoclinic, *P*2_1_/*c*	Melting point = 423–424 K
Hall symbol: -P 2ybc	Mo *K*α radiation, λ = 0.71073 Å
*a* = 14.1799 (2) Å	Cell parameters from 18727 reflections
*b* = 18.0877 (3) Å	θ = 2.1–35.0°
*c* = 16.6881 (2) Å	µ = 1.96 mm^−^^1^
β = 95.081 (1)°	*T* = 100 K
*V* = 4263.38 (11) Å^3^	Block, colorless
*Z* = 8	0.51 × 0.49 × 0.22 mm

### Data collection

**Table:**

Bruker APEXII CCD area-detector diffractometer	18727 independent reflections
Radiation source: sealed tube	12072 reflections with *I* > 2σ(*I*)
graphite	*R*_int_ = 0.044
φ and ω scans	θ_max_ = 35.0°, θ_min_ = 2.1°
Absorption correction: multi-scan (*SADABS*; Bruker, 2005)	*h* = −22→22
*T*_min_ = 0.437, *T*_max_ = 0.669	*k* = −29→27
85168 measured reflections	*l* = −26→25

### Refinement

**Table:**

Refinement on *F*^2^	Primary atom site location: structure-invariant direct methods
Least-squares matrix: full	Secondary atom site location: difference Fourier map
*R*[*F*^2^ > 2σ(*F*^2^)] = 0.038	Hydrogen site location: inferred from neighbouring sites
*wR*(*F*^2^) = 0.095	H-atom parameters constrained
*S* = 1.01	*w* = 1/[σ^2^(*F*_o_^2^) + (0.0378*P*)^2^ + 1.2763*P*] where *P* = (*F*_o_^2^ + 2*F*_c_^2^)/3
18727 reflections	(Δ/σ)_max_ = 0.003
549 parameters	Δρ_max_ = 0.50 e Å^−^^3^
0 restraints	Δρ_min_ = −0.45 e Å^−^^3^

### Special details

**Table:**

Experimental. The crystal was placed in the cold stream of an Oxford Cryosystems Cobra open-flow nitrogen cryostat (Cosier & Glazer, 1986) operating at 100.0 (1) K.
Geometry. All e.s.d.'s (except the e.s.d. in the dihedral angle between two l.s. planes) are estimated using the full covariance matrix. The cell e.s.d.'s are taken into account individually in the estimation of e.s.d.'s in distances, angles and torsion angles; correlations between e.s.d.'s in cell parameters are only used when they are defined by crystal symmetry. An approximate (isotropic) treatment of cell e.s.d.'s is used for estimating e.s.d.'s involving l.s. planes.
Refinement. Refinement of *F*^2^ against ALL reflections. The weighted *R*-factor *wR* and goodness of fit *S* are based on *F*^2^, conventional *R*-factors *R* are based on *F*, with *F* set to zero for negative *F*^2^. The threshold expression of *F*^2^ > σ(*F*^2^) is used only for calculating *R*-factors(gt) *etc*. and is not relevant to the choice of reflections for refinement. *R*-factors based on *F*^2^ are statistically about twice as large as those based on *F*, and *R*- factors based on ALL data will be even larger.

### Fractional atomic coordinates and isotropic or equivalent isotropic displacement parameters (Å^2^)

**Table:**

	*x*	*y*	*z*	*U*_iso_*/*U*_eq_	
Br1A	0.415133 (12)	0.399816 (9)	0.519413 (10)	0.02931 (5)	
O1A	0.93118 (7)	0.24881 (5)	0.28179 (6)	0.0205 (2)	
O2A	0.88259 (7)	0.02891 (5)	0.50720 (6)	0.01749 (19)	
O3A	0.79300 (9)	−0.21035 (6)	0.41508 (6)	0.0279 (2)	
O4A	0.74715 (8)	0.00621 (5)	0.24317 (6)	0.0216 (2)	
N1A	0.79667 (8)	0.25126 (6)	0.35033 (7)	0.0163 (2)	
N2A	0.99990 (10)	0.06683 (8)	0.24974 (8)	0.0274 (3)	
C1A	0.64576 (11)	0.33207 (8)	0.40150 (9)	0.0213 (3)	
H1AA	0.6868	0.3535	0.3677	0.026*	
C2A	0.57443 (11)	0.37470 (8)	0.42979 (10)	0.0244 (3)	
H2AA	0.5670	0.4240	0.4146	0.029*	
C3A	0.51453 (10)	0.34262 (8)	0.48100 (9)	0.0212 (3)	
C4A	0.52353 (10)	0.26904 (8)	0.50332 (9)	0.0210 (3)	
H4AA	0.4827	0.2483	0.5377	0.025*	
C5A	0.59440 (10)	0.22651 (8)	0.47372 (8)	0.0186 (3)	
H5AA	0.6002	0.1769	0.4878	0.022*	
C6A	0.65723 (10)	0.25764 (7)	0.42278 (8)	0.0166 (2)	
C7A	0.73374 (10)	0.21320 (7)	0.39098 (8)	0.0153 (2)	
C8A	0.74013 (10)	0.13710 (7)	0.40206 (8)	0.0163 (2)	
H8AA	0.6982	0.1130	0.4330	0.020*	
C9A	0.80937 (10)	0.09694 (7)	0.36680 (8)	0.0159 (2)	
C10A	0.87236 (10)	0.13553 (7)	0.32258 (8)	0.0166 (2)	
C11A	0.86418 (10)	0.21338 (7)	0.31897 (8)	0.0169 (3)	
C12A	0.81270 (9)	0.01486 (7)	0.37548 (8)	0.0157 (2)	
C13A	0.84560 (10)	−0.01815 (7)	0.44856 (8)	0.0161 (2)	
C14A	0.84073 (10)	−0.09404 (7)	0.45904 (9)	0.0181 (3)	
H14A	0.8641	−0.1157	0.5073	0.022*	
C15A	0.80025 (11)	−0.13720 (7)	0.39600 (9)	0.0199 (3)	
C16A	0.76879 (10)	−0.10705 (8)	0.32189 (9)	0.0195 (3)	
H16A	0.7434	−0.1367	0.2799	0.023*	
C17A	0.77663 (10)	−0.03050 (7)	0.31265 (8)	0.0173 (3)	
C18A	0.93260 (11)	0.32872 (8)	0.28667 (9)	0.0208 (3)	
H18A	0.9353	0.3446	0.3423	0.025*	
H18B	0.8761	0.3493	0.2580	0.025*	
C19A	1.01910 (12)	0.35363 (9)	0.24895 (10)	0.0294 (4)	
H19A	1.0228	0.4066	0.2506	0.044*	
H19B	1.0156	0.3372	0.1941	0.044*	
H19C	1.0744	0.3330	0.2780	0.044*	
C20A	0.92176 (10)	−0.00406 (8)	0.58126 (8)	0.0194 (3)	
H20A	0.9507	0.0336	0.6158	0.029*	
H20B	0.9685	−0.0401	0.5700	0.029*	
H20C	0.8722	−0.0277	0.6075	0.029*	
C21A	0.75721 (18)	−0.25954 (9)	0.35314 (11)	0.0464 (5)	
H21A	0.7560	−0.3089	0.3741	0.070*	
H21B	0.7973	−0.2580	0.3097	0.070*	
H21C	0.6942	−0.2449	0.3338	0.070*	
C22A	0.71499 (11)	−0.03616 (8)	0.17402 (9)	0.0233 (3)	
H22A	0.6994	−0.0036	0.1294	0.035*	
H22B	0.6598	−0.0639	0.1850	0.035*	
H22C	0.7641	−0.0695	0.1611	0.035*	
C23A	0.94346 (11)	0.09810 (8)	0.28221 (9)	0.0200 (3)	
Br1B	1.103317 (12)	0.395272 (9)	0.476239 (10)	0.02990 (5)	
O1B	0.57485 (7)	0.27568 (5)	0.71164 (6)	0.0202 (2)	
O2B	0.62698 (7)	0.02194 (5)	0.51718 (6)	0.01751 (19)	
O3B	0.73304 (9)	−0.19538 (6)	0.66989 (7)	0.0289 (3)	
O4B	0.76063 (8)	0.04510 (5)	0.78231 (6)	0.0212 (2)	
N1B	0.71233 (8)	0.26852 (6)	0.64794 (7)	0.0165 (2)	
N2B	0.47997 (10)	0.09979 (8)	0.73356 (9)	0.0300 (3)	
C1B	0.86314 (11)	0.34248 (8)	0.58904 (9)	0.0224 (3)	
H1BA	0.8191	0.3678	0.6168	0.027*	
C2B	0.93700 (12)	0.38133 (8)	0.55887 (10)	0.0255 (3)	
H2BA	0.9428	0.4321	0.5666	0.031*	
C3B	1.00145 (11)	0.34315 (8)	0.51734 (9)	0.0211 (3)	
C4B	0.99424 (10)	0.26760 (8)	0.50491 (9)	0.0212 (3)	
H4BA	1.0382	0.2429	0.4765	0.025*	
C5B	0.92041 (10)	0.22931 (8)	0.53546 (9)	0.0197 (3)	
H5BA	0.9150	0.1786	0.5274	0.024*	
C6B	0.85397 (10)	0.26631 (7)	0.57832 (8)	0.0161 (2)	
C7B	0.77575 (9)	0.22614 (7)	0.61271 (8)	0.0154 (2)	
C8B	0.76838 (10)	0.14930 (7)	0.61027 (8)	0.0159 (2)	
H8BA	0.8128	0.1216	0.5855	0.019*	
C9B	0.69472 (10)	0.11389 (7)	0.64472 (8)	0.0152 (2)	
C10B	0.62731 (10)	0.15791 (7)	0.67798 (8)	0.0163 (2)	
C11B	0.64068 (10)	0.23539 (7)	0.67824 (8)	0.0164 (2)	
C12B	0.69364 (9)	0.03151 (7)	0.65008 (8)	0.0156 (2)	
C13B	0.66365 (9)	−0.01382 (7)	0.58520 (8)	0.0151 (2)	
C14B	0.67260 (10)	−0.09074 (7)	0.58966 (9)	0.0181 (3)	
H14B	0.6503	−0.1205	0.5466	0.022*	
C15B	0.71545 (11)	−0.12171 (8)	0.65961 (9)	0.0205 (3)	
C16B	0.74559 (11)	−0.07822 (7)	0.72598 (9)	0.0194 (3)	
H16B	0.7735	−0.0998	0.7728	0.023*	
C17B	0.73323 (10)	−0.00229 (7)	0.72081 (8)	0.0165 (3)	
C18B	0.58732 (11)	0.35528 (7)	0.71198 (9)	0.0212 (3)	
H18C	0.5843	0.3740	0.6574	0.025*	
H18D	0.6483	0.3684	0.7392	0.025*	
C19B	0.50878 (13)	0.38711 (9)	0.75552 (11)	0.0322 (4)	
H19D	0.5140	0.4400	0.7567	0.048*	
H19E	0.5130	0.3685	0.8096	0.048*	
H19F	0.4490	0.3732	0.7282	0.048*	
C20B	0.59292 (10)	−0.02279 (8)	0.44918 (9)	0.0196 (3)	
H20D	0.5672	0.0086	0.4063	0.029*	
H20E	0.5446	−0.0557	0.4646	0.029*	
H20F	0.6443	−0.0511	0.4313	0.029*	
C21B	0.70011 (16)	−0.24452 (9)	0.60668 (11)	0.0396 (5)	
H21D	0.7188	−0.2941	0.6210	0.059*	
H21E	0.7270	−0.2306	0.5581	0.059*	
H21F	0.6323	−0.2419	0.5984	0.059*	
C22B	0.80585 (12)	0.01372 (9)	0.85449 (9)	0.0252 (3)	
H22D	0.8217	0.0523	0.8928	0.038*	
H22E	0.8625	−0.0115	0.8425	0.038*	
H22F	0.7637	−0.0207	0.8766	0.038*	
C23B	0.54596 (11)	0.12606 (8)	0.70986 (9)	0.0203 (3)	

### Atomic displacement parameters (Å^2^)

**Table:**

	*U*^11^	*U*^22^	*U*^33^	*U*^12^	*U*^13^	*U*^23^
Br1A	0.02877 (9)	0.03090 (8)	0.02875 (9)	0.01345 (6)	0.00520 (6)	−0.00364 (7)
O1A	0.0216 (5)	0.0169 (5)	0.0241 (5)	−0.0004 (4)	0.0076 (4)	0.0033 (4)
O2A	0.0201 (5)	0.0157 (4)	0.0159 (5)	0.0020 (4)	−0.0026 (4)	−0.0004 (4)
O3A	0.0481 (7)	0.0126 (5)	0.0226 (5)	−0.0021 (5)	0.0006 (5)	−0.0012 (4)
O4A	0.0311 (6)	0.0173 (5)	0.0156 (5)	0.0008 (4)	−0.0028 (4)	−0.0009 (4)
N1A	0.0176 (5)	0.0148 (5)	0.0165 (5)	0.0008 (4)	0.0013 (4)	0.0010 (4)
N2A	0.0293 (7)	0.0286 (7)	0.0250 (7)	0.0085 (6)	0.0069 (5)	0.0033 (5)
C1A	0.0248 (7)	0.0159 (6)	0.0235 (7)	0.0014 (5)	0.0039 (6)	0.0008 (5)
C2A	0.0286 (8)	0.0165 (6)	0.0285 (8)	0.0051 (6)	0.0044 (6)	−0.0014 (6)
C3A	0.0199 (7)	0.0230 (7)	0.0202 (7)	0.0063 (5)	−0.0006 (5)	−0.0048 (5)
C4A	0.0194 (7)	0.0254 (7)	0.0183 (7)	0.0040 (5)	0.0020 (5)	0.0016 (5)
C5A	0.0186 (7)	0.0180 (6)	0.0190 (7)	0.0024 (5)	0.0009 (5)	0.0021 (5)
C6A	0.0174 (6)	0.0156 (6)	0.0167 (6)	0.0012 (5)	0.0006 (5)	−0.0002 (5)
C7A	0.0169 (6)	0.0144 (6)	0.0144 (6)	0.0007 (5)	−0.0003 (5)	−0.0002 (5)
C8A	0.0178 (6)	0.0145 (6)	0.0167 (6)	0.0002 (5)	0.0022 (5)	−0.0001 (5)
C9A	0.0171 (6)	0.0137 (6)	0.0165 (6)	0.0012 (5)	−0.0005 (5)	−0.0006 (5)
C10A	0.0170 (6)	0.0170 (6)	0.0158 (6)	0.0022 (5)	0.0013 (5)	0.0008 (5)
C11A	0.0183 (6)	0.0164 (6)	0.0159 (6)	−0.0001 (5)	0.0012 (5)	0.0022 (5)
C12A	0.0160 (6)	0.0133 (6)	0.0181 (6)	0.0009 (5)	0.0028 (5)	−0.0002 (5)
C13A	0.0159 (6)	0.0146 (6)	0.0180 (6)	0.0013 (5)	0.0020 (5)	−0.0028 (5)
C14A	0.0212 (7)	0.0143 (6)	0.0187 (6)	0.0027 (5)	0.0009 (5)	0.0005 (5)
C15A	0.0246 (7)	0.0135 (6)	0.0222 (7)	0.0013 (5)	0.0046 (6)	−0.0011 (5)
C16A	0.0226 (7)	0.0162 (6)	0.0199 (7)	−0.0008 (5)	0.0017 (5)	−0.0049 (5)
C17A	0.0180 (6)	0.0167 (6)	0.0173 (6)	0.0025 (5)	0.0018 (5)	−0.0003 (5)
C18A	0.0240 (7)	0.0164 (6)	0.0221 (7)	−0.0024 (5)	0.0017 (6)	0.0029 (5)
C19A	0.0315 (9)	0.0280 (8)	0.0295 (8)	−0.0077 (7)	0.0068 (7)	0.0046 (7)
C20A	0.0203 (7)	0.0207 (6)	0.0168 (6)	0.0033 (5)	−0.0005 (5)	−0.0003 (5)
C21A	0.0914 (17)	0.0167 (7)	0.0298 (9)	−0.0114 (9)	−0.0025 (10)	−0.0053 (7)
C22A	0.0291 (8)	0.0252 (7)	0.0156 (7)	−0.0035 (6)	0.0007 (6)	−0.0022 (5)
C23A	0.0227 (7)	0.0186 (6)	0.0188 (6)	0.0022 (5)	0.0022 (5)	0.0036 (5)
Br1B	0.03256 (9)	0.03036 (8)	0.02790 (8)	−0.01565 (7)	0.00899 (7)	−0.00084 (6)
O1B	0.0215 (5)	0.0148 (4)	0.0251 (5)	0.0019 (4)	0.0066 (4)	−0.0036 (4)
O2B	0.0203 (5)	0.0152 (4)	0.0164 (5)	0.0000 (4)	−0.0017 (4)	0.0008 (4)
O3B	0.0466 (7)	0.0111 (4)	0.0266 (6)	−0.0007 (4)	−0.0092 (5)	0.0014 (4)
O4B	0.0309 (6)	0.0161 (5)	0.0159 (5)	0.0000 (4)	−0.0021 (4)	−0.0010 (4)
N1B	0.0180 (6)	0.0140 (5)	0.0174 (5)	0.0003 (4)	0.0013 (4)	−0.0017 (4)
N2B	0.0264 (7)	0.0298 (7)	0.0349 (8)	−0.0051 (6)	0.0099 (6)	−0.0027 (6)
C1B	0.0265 (8)	0.0163 (6)	0.0255 (7)	−0.0029 (5)	0.0077 (6)	−0.0017 (5)
C2B	0.0346 (9)	0.0158 (6)	0.0271 (8)	−0.0078 (6)	0.0072 (7)	−0.0023 (6)
C3B	0.0229 (7)	0.0207 (7)	0.0197 (7)	−0.0074 (5)	0.0018 (5)	0.0029 (5)
C4B	0.0198 (7)	0.0203 (7)	0.0238 (7)	−0.0013 (5)	0.0038 (6)	0.0014 (5)
C5B	0.0205 (7)	0.0158 (6)	0.0231 (7)	−0.0005 (5)	0.0031 (5)	0.0015 (5)
C6B	0.0185 (6)	0.0145 (6)	0.0152 (6)	−0.0010 (5)	−0.0001 (5)	0.0012 (5)
C7B	0.0172 (6)	0.0145 (6)	0.0146 (6)	−0.0004 (5)	0.0011 (5)	−0.0002 (5)
C8B	0.0169 (6)	0.0131 (5)	0.0178 (6)	0.0014 (5)	0.0022 (5)	0.0000 (5)
C9B	0.0166 (6)	0.0131 (6)	0.0157 (6)	−0.0002 (4)	0.0004 (5)	0.0009 (5)
C10B	0.0172 (6)	0.0154 (6)	0.0163 (6)	−0.0008 (5)	0.0017 (5)	−0.0005 (5)
C11B	0.0174 (6)	0.0151 (6)	0.0165 (6)	0.0014 (5)	0.0010 (5)	−0.0028 (5)
C12B	0.0163 (6)	0.0120 (5)	0.0189 (6)	−0.0007 (4)	0.0035 (5)	0.0002 (5)
C13B	0.0133 (6)	0.0151 (6)	0.0171 (6)	−0.0005 (4)	0.0022 (5)	0.0013 (5)
C14B	0.0202 (7)	0.0142 (6)	0.0196 (7)	−0.0022 (5)	0.0005 (5)	−0.0004 (5)
C15B	0.0246 (7)	0.0130 (6)	0.0238 (7)	−0.0016 (5)	0.0024 (6)	0.0021 (5)
C16B	0.0251 (7)	0.0146 (6)	0.0182 (6)	−0.0012 (5)	−0.0007 (5)	0.0033 (5)
C17B	0.0185 (6)	0.0141 (6)	0.0171 (6)	−0.0027 (5)	0.0022 (5)	−0.0007 (5)
C18B	0.0252 (7)	0.0143 (6)	0.0241 (7)	0.0021 (5)	0.0015 (6)	−0.0029 (5)
C19B	0.0352 (9)	0.0256 (8)	0.0371 (9)	0.0069 (7)	0.0102 (7)	−0.0078 (7)
C20B	0.0190 (7)	0.0203 (6)	0.0191 (7)	−0.0007 (5)	−0.0010 (5)	−0.0019 (5)
C21B	0.0672 (14)	0.0138 (7)	0.0347 (10)	−0.0012 (8)	−0.0124 (9)	−0.0030 (7)
C22B	0.0341 (9)	0.0239 (7)	0.0168 (7)	0.0015 (6)	−0.0025 (6)	0.0000 (6)
C23B	0.0219 (7)	0.0170 (6)	0.0221 (7)	0.0001 (5)	0.0030 (5)	−0.0034 (5)

### Geometric parameters (Å, °)

**Table:**

Br1A—C3A	1.9047 (14)	Br1B—C3B	1.9029 (14)
O1A—C11A	1.3426 (16)	O1B—C11B	1.3436 (16)
O1A—C18A	1.4478 (17)	O1B—C18B	1.4505 (17)
O2A—C13A	1.3662 (16)	O2B—C13B	1.3684 (16)
O2A—C20A	1.4389 (17)	O2B—C20B	1.4418 (17)
O3A—C15A	1.3668 (17)	O3B—C15B	1.3637 (17)
O3A—C21A	1.423 (2)	O3B—C21B	1.4259 (19)
O4A—C17A	1.3690 (17)	O4B—C17B	1.3669 (16)
O4A—C22A	1.4262 (17)	O4B—C22B	1.4310 (18)
N1A—C11A	1.3223 (17)	N1B—C11B	1.3185 (17)
N1A—C7A	1.3555 (17)	N1B—C7B	1.3550 (17)
N2A—C23A	1.1536 (19)	N2B—C23B	1.1504 (19)
C1A—C2A	1.387 (2)	C1B—C2B	1.392 (2)
C1A—C6A	1.3982 (19)	C1B—C6B	1.3940 (19)
C1A—H1AA	0.9300	C1B—H1BA	0.9300
C2A—C3A	1.385 (2)	C2B—C3B	1.380 (2)
C2A—H2AA	0.9300	C2B—H2BA	0.9300
C3A—C4A	1.385 (2)	C3B—C4B	1.385 (2)
C4A—C5A	1.3907 (19)	C4B—C5B	1.3894 (19)
C4A—H4AA	0.9300	C4B—H4BA	0.9300
C5A—C6A	1.4026 (19)	C5B—C6B	1.4021 (19)
C5A—H5AA	0.9300	C5B—H5BA	0.9300
C6A—C7A	1.4856 (19)	C6B—C7B	1.4833 (18)
C7A—C8A	1.3908 (18)	C7B—C8B	1.3942 (18)
C8A—C9A	1.3929 (18)	C8B—C9B	1.3921 (18)
C8A—H8AA	0.9300	C8B—H8BA	0.9300
C9A—C10A	1.3952 (19)	C9B—C10B	1.3962 (18)
C9A—C12A	1.4919 (18)	C9B—C12B	1.4930 (18)
C10A—C11A	1.4137 (19)	C10B—C11B	1.4141 (18)
C10A—C23A	1.4314 (19)	C10B—C23B	1.4333 (19)
C12A—C17A	1.3924 (19)	C12B—C13B	1.3943 (19)
C12A—C13A	1.4002 (19)	C12B—C17B	1.4017 (19)
C13A—C14A	1.3863 (18)	C13B—C14B	1.3985 (18)
C14A—C15A	1.392 (2)	C14B—C15B	1.386 (2)
C14A—H14A	0.9300	C14B—H14B	0.9300
C15A—C16A	1.389 (2)	C15B—C16B	1.394 (2)
C16A—C17A	1.3987 (19)	C16B—C17B	1.3862 (19)
C16A—H16A	0.9300	C16B—H16B	0.9300
C18A—C19A	1.497 (2)	C18B—C19B	1.498 (2)
C18A—H18A	0.9700	C18B—H18C	0.9700
C18A—H18B	0.9700	C18B—H18D	0.9700
C19A—H19A	0.9600	C19B—H19D	0.9600
C19A—H19B	0.9600	C19B—H19E	0.9600
C19A—H19C	0.9600	C19B—H19F	0.9600
C20A—H20A	0.9600	C20B—H20D	0.9600
C20A—H20B	0.9600	C20B—H20E	0.9600
C20A—H20C	0.9600	C20B—H20F	0.9600
C21A—H21A	0.9600	C21B—H21D	0.9600
C21A—H21B	0.9600	C21B—H21E	0.9600
C21A—H21C	0.9600	C21B—H21F	0.9600
C22A—H22A	0.9600	C22B—H22D	0.9600
C22A—H22B	0.9600	C22B—H22E	0.9600
C22A—H22C	0.9600	C22B—H22F	0.9600
			
C11A—O1A—C18A	117.22 (11)	C11B—O1B—C18B	116.81 (11)
C13A—O2A—C20A	116.84 (11)	C13B—O2B—C20B	117.63 (10)
C15A—O3A—C21A	117.74 (12)	C15B—O3B—C21B	118.15 (12)
C17A—O4A—C22A	118.45 (11)	C17B—O4B—C22B	117.36 (11)
C11A—N1A—C7A	117.82 (11)	C11B—N1B—C7B	118.22 (11)
C2A—C1A—C6A	121.37 (14)	C2B—C1B—C6B	121.14 (14)
C2A—C1A—H1AA	119.3	C2B—C1B—H1BA	119.4
C6A—C1A—H1AA	119.3	C6B—C1B—H1BA	119.4
C3A—C2A—C1A	118.86 (14)	C3B—C2B—C1B	118.81 (13)
C3A—C2A—H2AA	120.6	C3B—C2B—H2BA	120.6
C1A—C2A—H2AA	120.6	C1B—C2B—H2BA	120.6
C2A—C3A—C4A	121.46 (13)	C2B—C3B—C4B	121.72 (13)
C2A—C3A—Br1A	119.52 (11)	C2B—C3B—Br1B	119.39 (11)
C4A—C3A—Br1A	118.99 (11)	C4B—C3B—Br1B	118.90 (11)
C3A—C4A—C5A	119.23 (13)	C3B—C4B—C5B	119.01 (14)
C3A—C4A—H4AA	120.4	C3B—C4B—H4BA	120.5
C5A—C4A—H4AA	120.4	C5B—C4B—H4BA	120.5
C4A—C5A—C6A	120.73 (13)	C4B—C5B—C6B	120.76 (13)
C4A—C5A—H5AA	119.6	C4B—C5B—H5BA	119.6
C6A—C5A—H5AA	119.6	C6B—C5B—H5BA	119.6
C1A—C6A—C5A	118.34 (13)	C1B—C6B—C5B	118.55 (13)
C1A—C6A—C7A	120.16 (12)	C1B—C6B—C7B	119.93 (12)
C5A—C6A—C7A	121.49 (12)	C5B—C6B—C7B	121.52 (12)
N1A—C7A—C8A	122.09 (12)	N1B—C7B—C8B	121.72 (12)
N1A—C7A—C6A	115.98 (11)	N1B—C7B—C6B	116.01 (11)
C8A—C7A—C6A	121.94 (12)	C8B—C7B—C6B	122.26 (12)
C7A—C8A—C9A	120.00 (12)	C9B—C8B—C7B	120.24 (12)
C7A—C8A—H8AA	120.0	C9B—C8B—H8BA	119.9
C9A—C8A—H8AA	120.0	C7B—C8B—H8BA	119.9
C8A—C9A—C10A	118.08 (12)	C8B—C9B—C10B	117.84 (12)
C8A—C9A—C12A	119.70 (12)	C8B—C9B—C12B	119.73 (12)
C10A—C9A—C12A	122.20 (12)	C10B—C9B—C12B	122.30 (12)
C9A—C10A—C11A	117.83 (12)	C9B—C10B—C11B	118.04 (12)
C9A—C10A—C23A	121.53 (12)	C9B—C10B—C23B	121.34 (12)
C11A—C10A—C23A	120.63 (12)	C11B—C10B—C23B	120.62 (12)
N1A—C11A—O1A	120.15 (12)	N1B—C11B—O1B	119.99 (12)
N1A—C11A—C10A	123.97 (12)	N1B—C11B—C10B	123.84 (12)
O1A—C11A—C10A	115.87 (12)	O1B—C11B—C10B	116.16 (12)
C17A—C12A—C13A	118.45 (12)	C13B—C12B—C17B	117.99 (12)
C17A—C12A—C9A	120.45 (12)	C13B—C12B—C9B	122.99 (12)
C13A—C12A—C9A	120.94 (12)	C17B—C12B—C9B	118.71 (12)
O2A—C13A—C14A	123.15 (13)	O2B—C13B—C12B	115.69 (11)
O2A—C13A—C12A	115.80 (11)	O2B—C13B—C14B	122.72 (12)
C14A—C13A—C12A	121.05 (13)	C12B—C13B—C14B	121.58 (13)
C13A—C14A—C15A	118.79 (13)	C15B—C14B—C13B	118.55 (13)
C13A—C14A—H14A	120.6	C15B—C14B—H14B	120.7
C15A—C14A—H14A	120.6	C13B—C14B—H14B	120.7
O3A—C15A—C16A	124.25 (13)	O3B—C15B—C14B	124.23 (13)
O3A—C15A—C14A	113.65 (13)	O3B—C15B—C16B	114.31 (13)
C16A—C15A—C14A	122.08 (13)	C14B—C15B—C16B	121.46 (13)
C15A—C16A—C17A	117.68 (13)	C17B—C16B—C15B	118.77 (13)
C15A—C16A—H16A	121.2	C17B—C16B—H16B	120.6
C17A—C16A—H16A	121.2	C15B—C16B—H16B	120.6
O4A—C17A—C12A	114.60 (12)	O4B—C17B—C16B	123.28 (13)
O4A—C17A—C16A	123.52 (13)	O4B—C17B—C12B	115.13 (12)
C12A—C17A—C16A	121.85 (13)	C16B—C17B—C12B	121.57 (13)
O1A—C18A—C19A	106.54 (12)	O1B—C18B—C19B	106.73 (12)
O1A—C18A—H18A	110.4	O1B—C18B—H18C	110.4
C19A—C18A—H18A	110.4	C19B—C18B—H18C	110.4
O1A—C18A—H18B	110.4	O1B—C18B—H18D	110.4
C19A—C18A—H18B	110.4	C19B—C18B—H18D	110.4
H18A—C18A—H18B	108.6	H18C—C18B—H18D	108.6
C18A—C19A—H19A	109.5	C18B—C19B—H19D	109.5
C18A—C19A—H19B	109.5	C18B—C19B—H19E	109.5
H19A—C19A—H19B	109.5	H19D—C19B—H19E	109.5
C18A—C19A—H19C	109.5	C18B—C19B—H19F	109.5
H19A—C19A—H19C	109.5	H19D—C19B—H19F	109.5
H19B—C19A—H19C	109.5	H19E—C19B—H19F	109.5
O2A—C20A—H20A	109.5	O2B—C20B—H20D	109.5
O2A—C20A—H20B	109.5	O2B—C20B—H20E	109.5
H20A—C20A—H20B	109.5	H20D—C20B—H20E	109.5
O2A—C20A—H20C	109.5	O2B—C20B—H20F	109.5
H20A—C20A—H20C	109.5	H20D—C20B—H20F	109.5
H20B—C20A—H20C	109.5	H20E—C20B—H20F	109.5
O3A—C21A—H21A	109.5	O3B—C21B—H21D	109.5
O3A—C21A—H21B	109.5	O3B—C21B—H21E	109.5
H21A—C21A—H21B	109.5	H21D—C21B—H21E	109.5
O3A—C21A—H21C	109.5	O3B—C21B—H21F	109.5
H21A—C21A—H21C	109.5	H21D—C21B—H21F	109.5
H21B—C21A—H21C	109.5	H21E—C21B—H21F	109.5
O4A—C22A—H22A	109.5	O4B—C22B—H22D	109.5
O4A—C22A—H22B	109.5	O4B—C22B—H22E	109.5
H22A—C22A—H22B	109.5	H22D—C22B—H22E	109.5
O4A—C22A—H22C	109.5	O4B—C22B—H22F	109.5
H22A—C22A—H22C	109.5	H22D—C22B—H22F	109.5
H22B—C22A—H22C	109.5	H22E—C22B—H22F	109.5
N2A—C23A—C10A	178.83 (16)	N2B—C23B—C10B	178.29 (17)
			
C6A—C1A—C2A—C3A	0.9 (2)	C6B—C1B—C2B—C3B	0.4 (2)
C1A—C2A—C3A—C4A	−1.0 (2)	C1B—C2B—C3B—C4B	0.2 (2)
C1A—C2A—C3A—Br1A	−179.19 (12)	C1B—C2B—C3B—Br1B	−179.62 (12)
C2A—C3A—C4A—C5A	0.1 (2)	C2B—C3B—C4B—C5B	−0.3 (2)
Br1A—C3A—C4A—C5A	178.29 (11)	Br1B—C3B—C4B—C5B	179.45 (11)
C3A—C4A—C5A—C6A	1.0 (2)	C3B—C4B—C5B—C6B	0.0 (2)
C2A—C1A—C6A—C5A	0.1 (2)	C2B—C1B—C6B—C5B	−0.7 (2)
C2A—C1A—C6A—C7A	179.46 (14)	C2B—C1B—C6B—C7B	178.65 (14)
C4A—C5A—C6A—C1A	−1.1 (2)	C4B—C5B—C6B—C1B	0.5 (2)
C4A—C5A—C6A—C7A	179.61 (13)	C4B—C5B—C6B—C7B	−178.82 (13)
C11A—N1A—C7A—C8A	1.8 (2)	C11B—N1B—C7B—C8B	−2.1 (2)
C11A—N1A—C7A—C6A	−178.00 (12)	C11B—N1B—C7B—C6B	178.93 (12)
C1A—C6A—C7A—N1A	8.23 (19)	C1B—C6B—C7B—N1B	5.09 (19)
C5A—C6A—C7A—N1A	−172.45 (13)	C5B—C6B—C7B—N1B	−175.57 (13)
C1A—C6A—C7A—C8A	−171.55 (13)	C1B—C6B—C7B—C8B	−173.91 (14)
C5A—C6A—C7A—C8A	7.8 (2)	C5B—C6B—C7B—C8B	5.4 (2)
N1A—C7A—C8A—C9A	−3.9 (2)	N1B—C7B—C8B—C9B	−0.2 (2)
C6A—C7A—C8A—C9A	175.86 (13)	C6B—C7B—C8B—C9B	178.75 (13)
C7A—C8A—C9A—C10A	1.6 (2)	C7B—C8B—C9B—C10B	2.8 (2)
C7A—C8A—C9A—C12A	−176.91 (13)	C7B—C8B—C9B—C12B	−173.05 (13)
C8A—C9A—C10A—C11A	2.4 (2)	C8B—C9B—C10B—C11B	−3.1 (2)
C12A—C9A—C10A—C11A	−179.11 (13)	C12B—C9B—C10B—C11B	172.60 (13)
C8A—C9A—C10A—C23A	−177.84 (13)	C8B—C9B—C10B—C23B	175.86 (13)
C12A—C9A—C10A—C23A	0.6 (2)	C12B—C9B—C10B—C23B	−8.4 (2)
C7A—N1A—C11A—O1A	−176.51 (12)	C7B—N1B—C11B—O1B	−178.12 (12)
C7A—N1A—C11A—C10A	2.6 (2)	C7B—N1B—C11B—C10B	1.7 (2)
C18A—O1A—C11A—N1A	7.48 (19)	C18B—O1B—C11B—N1B	0.12 (19)
C18A—O1A—C11A—C10A	−171.71 (12)	C18B—O1B—C11B—C10B	−179.68 (12)
C9A—C10A—C11A—N1A	−4.7 (2)	C9B—C10B—C11B—N1B	1.0 (2)
C23A—C10A—C11A—N1A	175.50 (13)	C23B—C10B—C11B—N1B	−178.04 (13)
C9A—C10A—C11A—O1A	174.41 (12)	C9B—C10B—C11B—O1B	−179.24 (12)
C23A—C10A—C11A—O1A	−5.3 (2)	C23B—C10B—C11B—O1B	1.8 (2)
C8A—C9A—C12A—C17A	101.45 (16)	C8B—C9B—C12B—C13B	−79.57 (18)
C10A—C9A—C12A—C17A	−77.02 (18)	C10B—C9B—C12B—C13B	104.76 (16)
C8A—C9A—C12A—C13A	−73.84 (18)	C8B—C9B—C12B—C17B	93.84 (16)
C10A—C9A—C12A—C13A	107.69 (16)	C10B—C9B—C12B—C17B	−81.82 (17)
C20A—O2A—C13A—C14A	2.99 (19)	C20B—O2B—C13B—C12B	−178.53 (11)
C20A—O2A—C13A—C12A	−176.57 (11)	C20B—O2B—C13B—C14B	2.69 (18)
C17A—C12A—C13A—O2A	178.24 (12)	C17B—C12B—C13B—O2B	−178.96 (11)
C9A—C12A—C13A—O2A	−6.38 (19)	C9B—C12B—C13B—O2B	−5.51 (19)
C17A—C12A—C13A—C14A	−1.3 (2)	C17B—C12B—C13B—C14B	−0.2 (2)
C9A—C12A—C13A—C14A	174.04 (13)	C9B—C12B—C13B—C14B	173.29 (13)
O2A—C13A—C14A—C15A	178.92 (12)	O2B—C13B—C14B—C15B	176.43 (12)
C12A—C13A—C14A—C15A	−1.5 (2)	C12B—C13B—C14B—C15B	−2.3 (2)
C21A—O3A—C15A—C16A	4.8 (2)	C21B—O3B—C15B—C14B	−4.1 (2)
C21A—O3A—C15A—C14A	−176.65 (16)	C21B—O3B—C15B—C16B	176.73 (15)
C13A—C14A—C15A—O3A	−175.49 (13)	C13B—C14B—C15B—O3B	−176.30 (13)
C13A—C14A—C15A—C16A	3.1 (2)	C13B—C14B—C15B—C16B	2.8 (2)
O3A—C15A—C16A—C17A	176.75 (13)	O3B—C15B—C16B—C17B	178.31 (13)
C14A—C15A—C16A—C17A	−1.7 (2)	C14B—C15B—C16B—C17B	−0.9 (2)
C22A—O4A—C17A—C12A	175.92 (12)	C22B—O4B—C17B—C16B	1.3 (2)
C22A—O4A—C17A—C16A	−6.2 (2)	C22B—O4B—C17B—C12B	−176.93 (12)
C13A—C12A—C17A—O4A	−179.24 (12)	C15B—C16B—C17B—O4B	−179.75 (13)
C9A—C12A—C17A—O4A	5.36 (18)	C15B—C16B—C17B—C12B	−1.7 (2)
C13A—C12A—C17A—C16A	2.8 (2)	C13B—C12B—C17B—O4B	−179.59 (12)
C9A—C12A—C17A—C16A	−172.59 (13)	C9B—C12B—C17B—O4B	6.66 (18)
C15A—C16A—C17A—O4A	−179.09 (13)	C13B—C12B—C17B—C16B	2.2 (2)
C15A—C16A—C17A—C12A	−1.3 (2)	C9B—C12B—C17B—C16B	−171.56 (13)
C11A—O1A—C18A—C19A	173.84 (12)	C11B—O1B—C18B—C19B	−177.23 (13)

### Hydrogen-bond geometry (Å, °)

**Table:**

*D*—H···*A*	*D*—H	H···*A*	*D*···*A*	*D*—H···*A*
C1A—H1AA···N1A	0.93	2.45	2.7872 (19)	101
C1A—H1AA···O4B^i^	0.93	2.60	3.4813 (18)	159
C8A—H8AA···O2B	0.93	2.44	3.3391 (16)	162
C1B—H1BA···N1B	0.93	2.43	2.7751 (19)	102
C8B—H8BA···O2A	0.93	2.39	3.2889 (16)	162
C18A—H18B···O4B^i^	0.97	2.57	3.3360 (18)	136
C20A—H20B···O2A^ii^	0.96	2.58	3.2869 (17)	131
C20B—H20E···O2B^iii^	0.96	2.55	3.2169 (17)	126
C21A—H21B···O3B^iv^	0.96	2.57	3.154 (2)	119
C22A—H22B···N2B^iii^	0.96	2.58	3.479 (2)	155
C18B—H18D···Cg1^iv^	0.97	2.93	3.7798 (16)	147
C20A—H20C···Cg3	0.96	2.60	3.5075 (15)	157
C20B—H20F···Cg2	0.96	2.51	3.3845 (15)	152

Symmetry codes:  (i) *x*, −*y*+1/2, *z*−1/2; (ii) −*x*+2, −*y*, −*z*+1; (iii) −*x*+1, −*y*, −*z*+1; (iv) *x*, −*y*−1/2, *z*−1/2.
